# Role of the Cerebellum in Bipolar Disorder: A Systematic Literature Review

**DOI:** 10.7759/cureus.56044

**Published:** 2024-03-12

**Authors:** Hina Tai, Nermien Kandeel, Maya Menon, Andrew Ibrahim, Byeongyeon Choo, Rochell Santana, Ayodeji Jolayemi

**Affiliations:** 1 Medicine, St. George's University School of Medicine, St. George's, GRD; 2 Medicine, American University of Antigua, New York City, USA; 3 Medicine, Saba University School of Medicine, The Bottom, NLD; 4 Medicine, American University of Antigua, St. John’s, ATG; 5 Psychiatry, Interfaith Medical Center, New York City, USA

**Keywords:** cerebellar cognitive affective syndrome, cerebellar soft signs, neurological soft signs, schizophrenia and other psychotic disorders, bipolar disorders, cerebellum degeneration

## Abstract

The aim of this systematic literature review was to investigate the role of the cerebellum in the affective symptoms observed in patients with bipolar disorder. The present systematic literature review included clinical studies conducted from 2013-2023 among adult populations with bipolar I and II disorders, along with their specifiers. With regard to cerebellar pathology, it was found that those with bipolar disorder performed worse than their healthy counterparts in their ability to comprehend the mental states of others and in identifying negative mental states. Additionally, individuals with bipolar disorder had reduced gray matter loss in regions such as lobules I-IX, crus I, and crus II, different functional activation patterns of the thalamus, striatum, and hippocampus on functional magnetic resonance imaging (fMRI), and increased cortical thickness. Cerebro-cerebellar functional connectivities were altered in patients with bipolar disorder. The effects of lamotrigine and lithium on cerebellar volume and abnormalities are also discussed in this paper. The present systematic literature review illustrates the emerging involvement of the cerebellum in bipolar disorder and its affective symptoms and paves the way for future research and a better understanding of bipolar disorder.

## Introduction and background

Bipolar disorder is a complex psychiatric disorder characterized by recurrent episodes of mania and/or hypomania, followed by depression. Bipolar disorder has a lifetime prevalence between 1% and 5% [1]. Some evidence indicates bipolar disorder is inheritable, with studies demonstrating seven-fold increases in the lifetime risk of bipolar disease in those with a first-degree relative diagnosed with bipolar disorder [2]. Compared to the general population, bipolar disorder has a suicide rate 10-30 times higher, with up to 20% of people with untreated bipolar disorder ending their lives by suicide [1]. Consequently, patients with bipolar disorder have shorter life spans by nine to 17 years than the average population [1]. 

Neurological soft signs are deficits in motor coordination, sequencing of complex motor functions, and sensory integration that are not localized to a particular pathological lesion. Neurological soft signs have been established as features of both schizophrenia and bipolar disorder [3]. Cerebellar soft signs refer to a group of neurological deficits under the umbrella of neurological soft signs, which include deficits in posture, gait, kinetic functions, eye movements, and speech. Bipolar disorder patients demonstrate both cerebellar soft signs and neurological soft signs [4]. Chrobak et al. (2023) found that neurological soft signs and cerebellar soft signs worsened with the progression of bipolar disorder [4]. The authors suggested that neurological soft signs and cerebellar soft signs can be used as biological markers to assess the staging of bipolar disorder [4]. 

**Figure 1:**
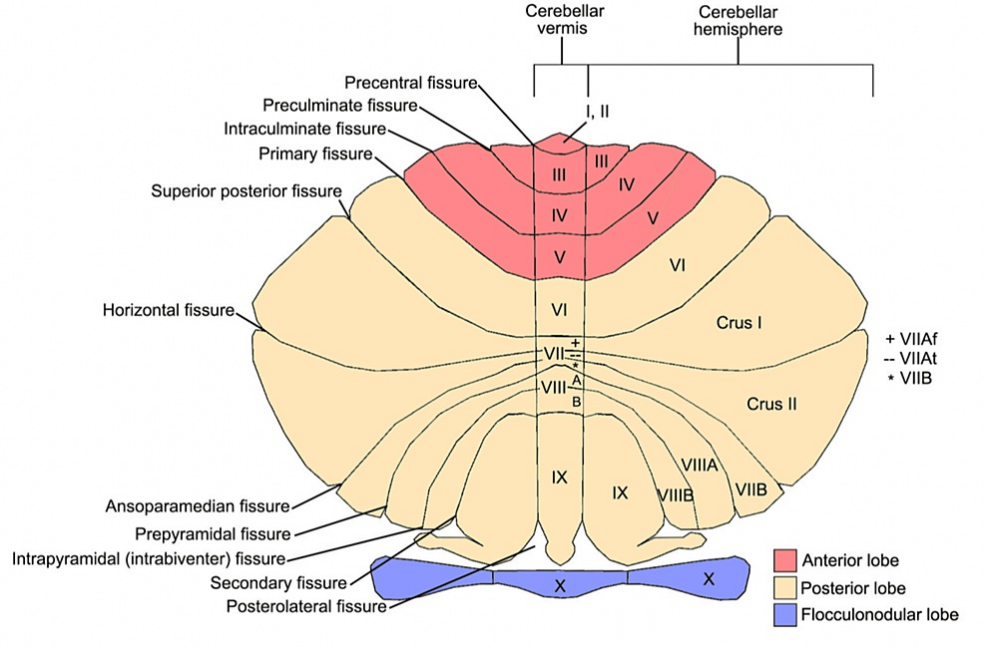
The anatomy of the anterior, posterior, and flocculonodular lobes of the cerebellum Reproduced with permission from D'Mello AM and Stoodley CJ [5]

More recently, research on bipolar disorder patients has indicated the involvement of the cerebellum in modulating not only motor but also affective symptoms observed in bipolar disorder. The cerebellum is divided into the anterior lobe (I-V) and the posterior lobe (VI, crus I, crus II, VII-IX). The anterior lobe is thought to be involved in motor function, while the posterior lobe is involved with emotional regulation, awareness, and high-order cognitive processing (Figure 1) [5]. 

Schmahmann and Sherman (1998) were the first to coin the term "cerebellar cognitive-affective syndrome" in a paper that examined 20 patients with diseases limited to the cerebellum [6]. The authors found that lesions to the posterior lobe of the cerebellum and vermis demonstrated behavioral changes as well as impairments in executive functioning [6]. The authors went on to define cerebellar cognitive affective syndrome as the following: (i) disturbances of executive function; (ii) impaired spatial cognition; (iii) personality change characterized by flattening or blunting of affect and disinhibited or inappropriate behavior; and (iv) linguistic difficulties [6]. 

Since Schmahmann and Sherman’s findings, several studies have gone on to report personality and behavioral changes after lesions to the cerebellum, and some studies have re-examined evidence dating back to the 19th century [7]. Hamilton et al. (1983) were among the earliest to state a link between cerebellar deficits and mood disorders after examining three cases of bipolar disorder and schizophrenia arising from cerebellar deficits (two due to cerebellar degeneration and one due to a cerebellar tumor) [8]. 

This novel understanding of the role of the cerebellum is not the mainstay and could be a significant barrier to treating patients with mood disorders such as bipolar disorder, depression, anxiety, and more. This paper aims to examine the current existing studies that have evaluated the relationship between bipolar disorder and the cerebellum, particularly the role of the cerebellum in emotional dysregulation in this population subset. Broadening the understanding of the role of the cerebellum in bipolar patients provides a new frontier for developing further interventions and treatments, as well as providing more effective care to patients. 

## Review

Methods

Eligibility Criteria

The criteria for eligibility included clinical studies and literature reviews centered on adult populations with bipolar I and II disorder diagnoses. Literature focusing on all subtypes, including psychotic features, seasonal patterns, and mixed features, was reviewed and included. The review synthesized research conducted within the last 10 years, between 2013 and 2023. Research studying the cerebellum's role in affective symptomatology and emotional regulation in bipolar disorder was included in the study. Retrospective cohort, prospective cohort, case-control, and cross-sectional studies were included in this study. 

Literature on pediatric populations, defined as those under the age of 18 years old, was excluded from the study. Research conducted before 2013 was excluded from the study. Research examining cerebellar changes in bipolar disorder relative to motor function was excluded. Studies that examined neuroimaging in bipolar disorder patients that did not specifically seek to examine changes in the cerebellum but rather the brain as a whole were excluded. Additionally, case reports and literature reviews were excluded from this review. 

Data Collection Process

The main database used to access peer-reviewed papers was PubMed. Search keywords such as “bipolar disorder and cerebellum,” “cerebellar signs bipolar disorder,” “neuroimaging bipolar disorder,” “affective symptoms and cerebellum,” “soft neurological signs bipolar disorder,” “cerebellum neuroimaging bipolar disorder," and “cerebellar cognitive affective syndrome” were used.

The search was filtered based on the date of publication as well as clinical studies conducted within the established time frame of the last 10 years. Research was identified by three independent reviewers to determine whether the study or publication met the inclusion criteria of the review. The reviewers then discussed any disagreements to determine whether to include the study in the final paper. 

**Figure 2:**
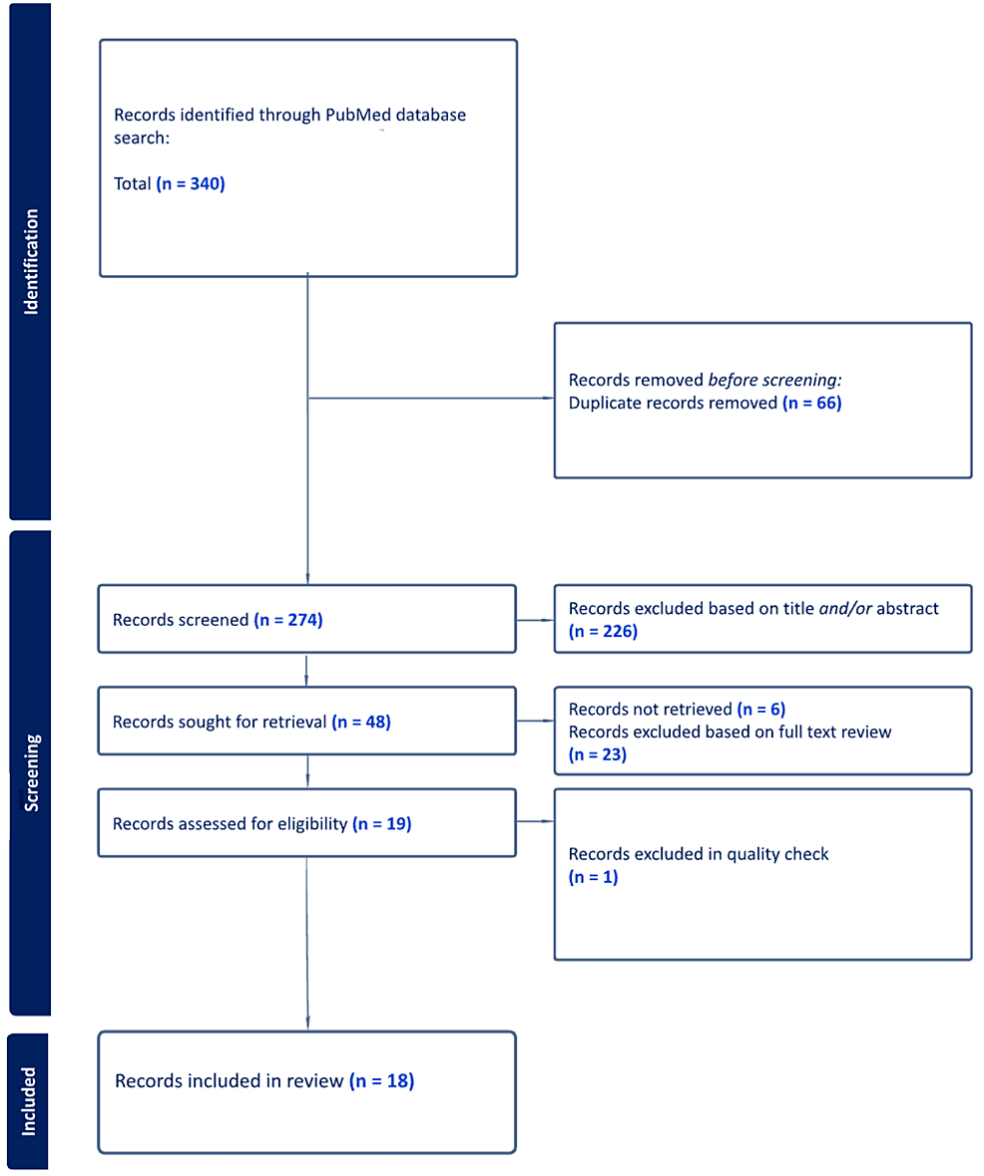
The PRISMA flowchart showing the study selection process PRISMA: Preferred Reporting Items for Systematic Reviews and Meta-Analysis

An initial search using the keywords was conducted on PubMed, generating 340 publications, which were then extracted and uploaded to an electronic database. Duplicated studies were removed, leaving 274 studies requiring screening for inclusion and exclusion. Three reviewers independently screened the title and abstract, discussing any arising conflicts to reach a consensus. After this initial screening, 42 studies were determined to be eligible for inclusion, which were then reviewed based on the full article text. Finally, 24 studies were excluded for not meeting the inclusion criteria after reviewing the full text, and 18 studies remained and were analyzed for inclusion in the current literature review. We were unable to find six full-text articles, which were excluded from the study. A flow chart of the data collection process after incorporating the inclusion and exclusion criteria is demonstrated in Figure 2. 

Results 

The final study found 18 studies that met the inclusion criteria, resulting in a total sample size of 3,068 analyzed in this literature review. This included 1,100 bipolar disorder patients (35.85%). This bipolar group was further divided into 524 unspecified bipolar disorder patients (17.08%), 448 bipolar I disorder patients (14.60%), and 128 bipolar II disorder patients (4.17%). Additionally, the study included 463 individuals diagnosed with schizophrenia (15.09%), 32 participants with cerebellar neurodegenerative disorders (1.04%), and 1,473 healthy controls (48.01%). 

All of the studies analyzed in this review utilized functional magnetic resonance imaging (fMRI) imaging diagnostics in their measurement assessments. Participants were additionally evaluated with a range of psychological measures, including the Questionnaire of Cognitive and Affective Empathy, the Wechsler Adult Intelligence Test-Revised, the Sequence Test, and the Faux Pas test. None of these tests were utilized in more than one study. A summary of the reviewed papers is included in Table 1. 

**Table 1:** Summary of the included studies

Author	Title	Study design	Population	Findings
Olivito et al. (2022) [9]	Theory of Mind Profile and Cerebellar Alterations in Remitted Bipolar Disorder 1 and 2: A Comparison Study	Cross-sectional study	17 participants with a diagnosis of bipolar I disorder, 13 participants with bipolar II disorder, and 77 healthy controls	Voxel-based morphometry showed a reduction in gray matter density in the anterior and posterior portions of the cerebellum, with a preference for the right hemisphere in patients with bipolar I disorder. In patients with bipolar II disorder, voxel-based morphometry showed a diffuse pattern of gray matter reduction in the cerebellum. The researchers also found that the decrease in gray matter volumes in bipolar I and II disorder patients was correlated with low scores on the Theory of the Mind assessments.
Lupo et al. (2021) [10]	Comparison of Cerebellar Grey Matter Alterations in Bipolar and Cerebellar Patients: Evidence from Voxel-Based Analysis	Cross-sectional study	29 patients with bipolar disorder, 32 subjects with cerebellar neurodegenerative pathologies, and 37 age-matched healthy subjects	The present study demonstrated that bipolar disorder patients and patients with cerebellar degenerative disorders showed a common pattern of loss in cerebellar lobules that are involved in cognitive and emotional abilities, such as crus I and crus II. The changes observed in these cerebellar regions may potentially be the underlying neuroanatomical key that contributes to the manic symptoms seen in bipolar disorder, as well as the mood disturbances seen in patients with cerebellar diseases.
Shaffer Jr. et al. (2018) [11]	Impaired Sensory Processing Measured by Functional MRI in Bipolar Disorder Manic and Depressed Mood States	Cross-sectional study	40 participants with bipolar I disorder and 33 healthy controls	The bipolar patient group had a decreased functional response in bilateral visual areas and cortical areas, including the cuneal cortex and occipital pole, when compared to healthy controls. No significant difference in functional activity between the euthymic group and healthy controls was observed. Furthermore, no significant difference in functional activity between bipolar-depressed patients and bipolar-manic patients was seen. There was decreased functional activity in numerous visual areas, subcortical brain regions, and cerebellar vermis in the depressed and manic bipolar groups compared to healthy controls. The manic group also showed decreased functional activity in the cerebellar vermis and adjacent left posterior lobe of the cerebellum when compared to the euthymic group. There were more false positives when identifying red squares on the checkerboard between the bipolar group and healthy controls. No differences in latency, hits, or misses were observed.
Kim et al. (2020) [12]	Changes in Cortical Thickness and Volume of Cerebellar Subregions in Patients With Bipolar Disorders	Cross-sectional study	90 patients with bipolar disorder and 166 healthy participants in Korea	Widespread and significant cortical thickening in all the cerebellar subregions was observed. These results provide evidence of the involvement of the cerebellum in bipolar disorder.
Kim et al. (2013) [13]	Posterior Cerebellar Vermal Deficits in Bipolar Disorder	Cross-sectional study	49 bipolar disorder patients (24 medication-naive and 25 medication-treated) and 50 matched healthy controls	The current findings suggested that bipolar disease-related deficits in the posterior cerebellar regions, which appeared to progress over the course of the illness, could potentially be ameliorated by proper treatment with mood stabilizers.
Cui et al. (2022) [14]	Altered Cerebellar Gray Matter and Cerebellar-Cortex Resting-State Functional Connectivity in Patients With Bipolar Disorder I	Cross-sectional study	45 patients with bipolar I disorder and 40 healthy controls from Guangzhou, China	Bipolar I disorder patients showed decreased cerebellar gray matter volume and disrupted cerebellar-cortex resting-state functional connectivity. This suggests that cerebellar abnormalities may play an important role in the pathogenesis of bipolar I disorder.
Lapomarda et al. (2021) [15]	Out of Control: An Altered Parieto-Occipital-Cerebellar Network for Impulsivity in Bipolar Disorder	Cross-sectional study	187 participants were initially selected from a shared neuroimaging dataset found on the OpenNeuro project from the UCLA Consortium for Neuropsychiatric Phenomics; 46 patients with bipolar I disorder and 60 healthy participant controls.	Decreased gray matter concentration (GMC) in independent component 14 (IC14), a parietal-occipital and cerebellar network designated by the Group ICA of the fMRI Toolbox (GIFT) software, in patients with bipolar disorder compared with healthy controls. This network includes the precuneus and inferior parietal lobule and numerous cerebellar regions, including the tonsil, declive, pyramis, tuber, and uvula. Lower GMC in IC14 is associated with higher attentional impulsiveness, motor impulsiveness, and non-planning impulsiveness. High self-reported measures of attentional impulsiveness, non-planning impulsiveness, and motor impulsiveness were found in patients with bipolar disorder compared to healthy controls, but no difference in risk-taking behavior as assessed with Balloon Analogue Risk Task (BART) was observed. The GMC in IC14 was not correlated with increased risk-taking behavior.
Laidi et al. (2015) [16]	Cerebellar Volume in Schizophrenia and Bipolar I Disorder With and Without Psychotic Features	Cross-sectional study	115 bipolar I disorder patients, 32 patients with schizophrenia, and 52 healthy controls	A reduction in cerebellar cortical volume was specific to schizophrenia. Cerebellar dysfunction in bipolar disorder, if present, appeared to be more subtle than a reduction in cerebellar volume.
Moussa-Tooks et al. (2022) [17]	Cerebellar Structure and Cognitive Ability in Psychosis	Cross-sectional study	249 schizophrenia spectrum, 108 bipolar with psychotic features, 217 nonpsychiatric control	Low premorbid cognitive functioning was associated with smaller whole and regional cerebellum volumes, including cognitive and motor regions, in psychosis.
Johnson et al. (2018) [18]	Alterations of the Cerebellum and Basal Ganglia in Bipolar Disorder Mood States Detected by Quantitative T1ρ Mapping	Cross-sectional study	40 patients with bipolar I disorder and 29 healthy controls matched for age and gender	Increased T1ρ relaxation time clusters across multiple cerebellar regions, including the right and left cerebellar lobules V, VI, VIII, IX, right lobule VII, and cerebellar white matter, were noted in bipolar patients in the euthymic, depressed, and manic mood states when compared to healthy controls. Increased T1ρ cerebellar relaxation times were observed in bipolar patients in a depressed state compared to euthymic patients. There was no difference between euthymic and manic cerebellar T1ρ relaxation times.
Bauer et al. (2018) [19]	Changes in Amygdala, Cerebellum, and Nucleus Accumbens Volumes in Bipolar Patients Treated With Lamotrigine	Non-randomized experimental study	12 patients with bipolar II disorder and 12 healthy controls	No statistically significant difference was found between cerebellar volumes of bipolar disorder lamotrigine responders, nonresponders, and healthy controls. Bipolar disorder lamotrigine responders had a greater likelihood of having decreased amygdala, cerebellum, and nucleus accumbens volumes following 12-week lamotrigine treatment. Non-responders had an increased likelihood of displaying increased cerebellum, amygdala, and nucleus accumbens volumes.
Laidi et al. (2019) [20]	Cerebellar Parcellation in Schizophrenia and Bipolar Disorder	Cross-sectional study	182 patients with schizophrenia; 144 patients with bipolar disorder; and 322 controls	Patients with schizophrenia showed a smaller global cerebellar gray matter volume compared to controls. The authors did not find alterations in the cerebellum in patients with bipolar disorder; however, patients medicated with lithium had a larger size of the anterior cerebellum compared to patients not treated with lithium.
Shinn et al. (2017) [21]	Aberrant Cerebellar Connectivity in Bipolar Disorder With Psychosis	Cross-sectional study	49 patients with bipolar disorder with a lifetime history of psychotic features and 55 healthy controls	Compared to the healthy controls, the participants with bipolar disorder with psychotic features had decreased cerebro-cerebellar functional connectivity. Specifically, they found decreased functional connectivity in the following cerebellar anatomical regions: V, VI, VIIb, VIIIa, and crus I-II. These anatomical regions correlate to the following networks: somatomotor A, salience, ventral attention, and frontoparietal control A and B.
Siciliano et al. (2023) [22]	The Role of the Cerebellum in Sequencing and Predicting Social and Non-social Events in Patients With Bipolar Disorder	Cross-sectional study	18 patients diagnosed with bipolar disorder in the euthymic phase, 24 healthy participants in control group 1, and 37 healthy subjects with previous MRI data in control group 2	There was a significant reduction in behavioral sequences and spatial sequences on the sequences test in the bipolar group compared to healthy controls. There was no significant difference in verbal sequences. On the Faux Pas Test, the bipolar group had significantly lower total scores than healthy controls when assessing "faux pas" stories and cognitive components of the test. No difference in "no-faux pas stories" or affective components of the Faux Pas Test was seen between groups. Two large clusters of gray matter reduction were noted in the cerebellar regions of bipolar disorder patients compared to healthy controls. Cluster one was noted in right crus II, and cluster 2 was noted in right lobules I-IV and V with extension to lobule VI, crus I, VIIIa, VIIIb, IX, left crus I and II, vermis crus II, vermis VI, and vermis VIIIa.
Chen et al. (2019) [23]	Abnormal Cerebellum-DMN Regions Connectivity in Unmedicated Bipolar II Disorder	Cross-sectional study	90 patients with unmedicated bipolar II disorder in depressive episodes and 100 healthy controls from Guangzhou, China	Increased connectivity between cerebellar right crus I and the bilateral precuneus, a posterior default mode network association area. Decreased connectivity between left cerebellar crus II and bilateral medial prefrontal cortex; decreased connectivity between left cerebellar crus II and right medial frontal gyrus, anterior default mode network association areas.
Olivito et al. (2022) [24]	Aberrant Cerebello-Cerebral Connectivity in Remitted Bipolar Patients 1 and 2: New Insight into Understanding the Cerebellar Role in Mania and Hypomania	Cross-sectional study	17 euthymic patients with bipolar 1 disorder and 13 euthymic patients with bipolar 2 disorder vs. 37 sex- and age-matched healthy subjects	Impaired cerebro-cerebellar connectivity is related to the manic and hypomanic states of bipolar disorder. These altered functional connectivity patterns persist during euthymia, supporting the hypothesis that cerebro-cerebellar functional connectivity changes reflect the neural correlate of subthreshold symptoms, a compensatory mechanism to maintain a state of euthymia.
Liang et al. (2022) [25]	Altered Empathy-Related Resting-State Functional Connectivity in Patients With Bipolar Disorder	Cross-sectional study	37 patients with bipolar disorder vs. 42 healthy controls in Beijing, China	The correlations between cognitive empathy and the resting state of functional connectivity were weaker in bipolar disorder patients than in healthy controls.
Saleem et al. (2023) [26]	Functional Connectivity of the Cerebellar Vermis in Bipolar Disorder and Associations with Mood	Cross-sectional study	128 participants with bipolar I disorder and 83 healthy controls	The connectivity of the vermis was found to be greater in bipolar disorder to regions involved in motor control and emotion, while reduced connectivity was observed in a region associated with language production.

Cerebellar Pathologies and Bipolar Disorder 

A study done by Olivito et al. (2022) investigated the Theory of Mind profile and the potential of cerebellar contributions in patients with bipolar I and II disorders [9]. The Theory of Mind can be defined as the capacity to comprehend the mental states of others, and to understand and explain their behaviors. The authors found that the bipolar I and II disorder groups had significantly lower scores in the Faux Pas test compared to the healthy group [9]. The Faux Pas test was utilized by the researchers to evaluate the Theory of Mind. More specifically, the authors found that the bipolar I and II disorder groups had significantly lower scores in the cognitive component of the Faux Pas test as compared to the healthy group [9]. Additionally, they found that the bipolar II disorder group did significantly worse compared to the healthy group in the affective component of the theory of the mind assessment [9]. This was an assessment used by the authors to evaluate the participants’ ability to understand the behaviors and mental states of those around them [9]. Moreover, it was found that patients with bipolar II disorder scored significantly less in the Reading the Mind in the Eyes test when attempting to detect negative mental states, such as worry [9]. The Reading the Mind in the Eyes test was used by the researchers to assess how well participants were able to determine the positive and negative mental states of people in photographs based solely on their gaze [9]. On MRI, patients with bipolar I disorder had reduced gray matter density in the anterior and posterior aspects of the cerebellum, right lobules I-IV, VI, crus I and II, left crus I and II, and vermis crus II, V, and VIIIa [9]. Bipolar II disorder patients showed diffusely decreased gray matter density, as well as right lobules I-IV, V, crus I and II, left crus II, and vermis crus II [9]. 

Another study found that, through voxel-based analysis, the bipolar group and the cerebellar neurodegenerative disorders group had a statistically significant loss of gray matter in the cerebellar cortex compared to the healthy control group [10]. In the bipolar disorder group, gray matter volume loss in the right lobules I-V, crus I, VIIb, IX, vermis crus II, left lobule VI, and bilateral crus II was seen when compared to the healthy group [10]. Moreover, in the cerebellar neurodegenerative disorders group, gray matter volume loss in the bilateral lobules I-IV, bilateral lobule VI, right V, and bilateral crus I and crus II was seen when compared to the healthy group [10]. Finally, there was an overlap in gray matter reduction in the bipolar disorder group and the cerebellar neurodegenerative disorders group, which included right lobule V, right crus I, and bilateral crus II [10].

A neuroimaging study by Shaffer et al. (2018) was conducted to compare functional activation patterns in bipolar disorder patients across euthymic, manic, and depressive mood states using a flashing checkerboard stimulus while under fMRI [11]. It was found that patients with bipolar disorder who were in either manic or depressive mood states had decreased functional activity in the cerebellar vermis compared to healthy controls [11]. The manic bipolar group also demonstrated decreased functional activity compared to the euthymic bipolar group in the cerebellar vermis, which included areas adjacent to the left posterior cerebellar lobe [11]. Differences were also noted in the functional activation patterns of the thalamus, striatum, hippocampus, right supramarginal gyrus, and left posterior forceps in bipolar mood states and healthy controls [11]. Depressed and manic mood states were also associated with decreased functional activation of the bilateral visual cortices compared to healthy control groups [11]. The bipolar disorder group also demonstrated increased false positives while identifying red squares on the checkerboard stimulus compared to healthy controls [11]. The authors of the study suggest altered visual processing may be a feature of bipolar mood states, leading to a tendency towards disinhibition or hyperactivity [11]. 

Kim et al. (2020) aimed to investigate the differences in cerebellar volume and cortical thickness in patients with bipolar disorder [12]. The findings revealed that patients with bipolar disorder had significantly greater cortical thickness in all subregions compared to healthy controls [12]. However, there was no significant difference in overall cerebellar volume between the two groups, except for a decreased volume in the left lobule IX of patients with bipolar disorder [12].

A neuroimaging study investigated the structural deficits in the cerebellum of individuals with bipolar disorder using magnetic resonance imaging and voxel-based morphometry [13]. The study revealed significant reductions in gray matter density in the posterior-inferior vermal regions (lobules VIII and IX) and the right posterior cerebellar hemispheric region (crus I) of bipolar disorder patients [13]. These deficits were localized to the cerebellum [13]. The study also explored the relationships between cerebellar deficits, disease duration, and medication treatment [13]. The results suggested longer illness duration associated with increased cerebellar deficits, while the progression of these deficits may attenuate or slow the progression of the disease with medication treatment [13].

Neuroimaging of the Cerebellum in Bipolar Disorder

Cerebellar anatomical abnormalities in bipolar disorder: Cui et al. (2022) aimed to investigate changes in gray matter volume in the brain and functional connectivity in patients with bipolar disorder I [14]. The researchers used voxel-based morphometry analysis to compare gray matter volume between bipolar I disorder patients and healthy controls [14]. They found that bipolar disorder patients had significantly decreased gray matter volume in the left lobules V and VI of the cerebellum [14]. Additionally, the researchers performed functional connectivity analysis [14]. They found that bipolar patients had decreased functional connectivity between the cerebellum and several brain regions, including the bilateral superior temporal gyrus, bilateral insula, bilateral rolandic operculum, right putamen, and left precentral gyrus [14]. These regions are involved in emotional processing, social cognition, auditory processing, and reward circuitry [14]. The findings suggest that disrupted functional connectivity between the cerebellum and these regions may be involved in the pathogenesis of bipolar disorder I [14]. Overall, the study suggests that abnormalities in the cerebellum, specifically in the left lobule V and lobule VI, may be associated with bipolar I disorder and its related motor, emotional, and cognitive symptoms [14].

Lapomarda et al. (2021) aimed to identify neurostructural markers for impulsivity in patients with bipolar disorder [15]. They used MRI images of the brain in both individuals with bipolar disorder and those of healthy controls [15]. Using source-based morphometry, researchers found the independent component 14 (ICI4) network to be a significant predictor of bipolar disorder [15]. Patients had a smaller gray matter concentration in the ICI4 network region [15]. This network included portions of the cerebellum and showed alterations in volume in bipolar disorder patients compared to healthy controls [15]. The study also assessed impulsivity and risky behavior using self-report measures and performance-based tasks [15]. Bipolar disorder patients were found to have higher scores on impulsivity measures compared to healthy controls, but there were no differences in risky behavior [15]. There were negative correlations between gray matter concentration in IC14 and impulsiveness measures, indicating that lower gray matter concentration in this network was associated with greater impulsivity [15]. 

Laidi et al. (2015) investigated cerebellar volume abnormalities using MRI imaging to measure both the outer cortex and inner white matter volume in individuals with bipolar I disorder, schizophrenia, and healthy controls [16]. The study did not compare the findings between schizophrenia and bipolar I disorder and focused on comparing them separately [16]. The findings of the study revealed that patients with bipolar I disorder had significantly reduced cerebellar cortex volume compared to healthy controls [16]. However, there was no significant difference in the deeper cerebellar white matter volumes [16]. This study supports the idea that there may be structural abnormalities and a reduction in the volume of the cerebellum in individuals with bipolar I disorder, particularly in the cortex [16].

According to Moussa-Tooks et al. (2022), there was no significant cerebellar structural difference between schizophrenic spectrum patients, bipolar with psychotic features, and healthy controls [17]. The authors hypothesized that developmental risk factors are the most profound element causing cerebellar structural abnormalities seen in psychotic patients [17]. 

A study utilizing T1𝛒 mapping, a magnetic resonance imaging technique used to measure metabolism markers in euthymic, depressed, and manic mood states, found increased T1𝛒 relaxation time in the cerebellum in all three mood states [18]. The authors suggest this could be due to reduced pH resulting from underlying metabolism dysfunction, lending support to the cerebellar metabolic dysfunction hypothesis in bipolar disorder [18]. In particular, the study found T1𝛒 differences in lobule VII of participants with mania and lobules VI and VII of participants in euthymic and depressed states [18]. Euthymic and depressed groups also had altered T1𝛒 signal in aspects of lobules IV and/or V, suggesting differences in motor control sites “and supports previous observations of altered cerebellar activity during motor tasks in bipolar disorder” [18]. These findings lend support to the hypothesis that cerebellar metabolic dysfunction in both motor and emotional processing regions contributes to the pathophysiology of bipolar disorder [18]. 

Bauer et al. (2018) examined the effects of lamotrigine therapy on cerebral and limbic brain structures in patients with bipolar II disorder using T1-weighted MRI imaging [19]. Participants with bipolar II disorder who responded to lamotrigine demonstrated decreased volumes in the amygdala, cerebellum, hippocampus, and nucleus accumbens [19]. In contrast, bipolar II disorder patients who were designated as non-responders to lamotrigine exhibited an increased volume in the amygdala, cerebellum, and nucleus accumbens [19]. These results suggest that lamotrigine exerts its effects on bipolar II disorder by preventing cerebral and cerebellar hypertrophy [19]. 

Laidi et al. (2019) examined the effect of lithium on cerebellar anatomy in bipolar disorder and schizophrenia using 3 Tesla magnetic resonance imaging (3T MRI) [20]. The study found no alterations in the cerebellum in bipolar disorder patients; however, they did find patients medicated with lithium had a larger size of the anterior cerebellum compared to patients not treated with lithium [20]. According to the authors, the anterior cerebellum is connected to the sensorimotor cortex, which may explain why patients medicated with lithium display cerebellar motor syndrome [20]. However, the study found no effects of lithium in the posterior cerebellum, while abnormalities were seen in schizophrenia patients [20]. Laidi et al. concluded that “it is unlikely the absence of cerebellar changes in bipolar disorder was related to the neuroprotective effects of lithium” [20]. 

Another study found no significant differences when comparing T1𝛒 maps voxel-wise in patients using lithium and patients not using lithium, “suggesting that lithium use did not have a normalizing effect on T1𝛒 when evaluated across mood states” [18]. 

Cerebellar functional connectivities in bipolar disorder: One study examined the functional connectivity between the cerebellar network and the cerebral network in patients with bipolar disorder with psychotic features and how this compared to their healthy control counterparts [21]. The researchers found that, compared to the healthy controls, the participants with bipolar disorder with psychotic features had decreased cerebro-cerebellar functional connectivity [21]. Specifically, they found decreased functional connectivity in the following cerebellar anatomical regions: V, VI, VIIb, VIIIa, and crus I-II [21]. These anatomical regions correlate to the following networks: somatomotor A, salience, ventral attention, and frontoparietal control A and B [21]. This illustrates the various circuitries that are disrupted in bipolar disorder, contributing to altered cognitive processing and the inability to sustain attention, as reflected in these patients [21]. 

Siciliano et al. (2023) examined the differences in cerebellar gray matter alterations in patients with bipolar disorder compared to healthy controls using voxel-based morphometry [22]. Two large clusters of gray matter reduction were noted between bipolar disorder and healthy control groups [22]. Cluster one comprised the right crus II, while cluster II comprised the right lobules I-IV and V with extension to lobule VI, crus I, VIIIa, VIIIb, IX, left crus I and II, vermis crus II, vermis VI, and vermis VIIIa [22]. This study also assessed participants using the Faux Pas test, a series of 20 stories where participants had to detect if a social faux pas had occurred [22]. This test is used to assess one's ability to make inferences about another person's state of mind. The authors found that the bipolar group had decreased detection of “social faux pas” and the ability to “justify other people’s behaviors” when they were identified compared to healthy controls, but no differences in “no social pas” detection [22]. The authors suggest that the cerebellum functions to sequence and predict events in both sensorimotor and cognitive domains and detect deviations from “correct” sequences [22]. The authors speculate that decreased gray matter in posterior cerebellar areas hinders the cerebellum's ability to modulate the effects of the neocortex, which may play a foundational role in the pathophysiology of bipolar disorder [22].

Chen et al. (2019), while examining the functional connectivity of the cerebellum-default mode network in unmedicated bipolar II disorder patients, found increased connectivity in the right crus I and bilateral precuneus and decreased connectivity between the left crus II and right medial frontal gyrus [23]. Chen et al. believe the disrupted connectivity of the left cerebellar crus II-medial frontal gyrus may contribute to the rumination symptoms in bipolar II disorder depression, and “increased functional connectivity of the cerebellar-precuneus could be a compensatory response to structural deficits of the precuneus in bipolar disorder” [23]. 

Olivito et al. (2022) have studied the functional connectivity of brain areas among patients who were diagnosed with bipolar I and II disorder using T1-weighted and resting-state functional connectivity scans [24]. It was found that different patterns of cerebro-cerebellar changes led to impaired cerebellar action on cerebral regions, which could act as a compensatory mechanism to maintain a state of clinical remission of hypomanic or manic symptoms [24]. These alterations in resting state functional connectivity play a role in the manifestation of hypomanic or manic symptoms in bipolar I and II disorder patients compared to healthy controls [24]. 

Liang et al. (2022) aimed to understand differences in cognitive and affective empathy by examining the empathy-related resting-state functional connectivity in bipolar patients compared to healthy controls by using self-reported Questionnaires of Cognitive and Affective Empathy, the Yoni behavioral task, and resting-state fMRI brain scans [25]. With regard to affective empathy, the authors did not find a significant correlation in either healthy controls or bipolar disorder patients [25]. However, there were significant positive correlations between cognitive empathy and the resting-state functional connectivity between the dorsal medial prefrontal cortex and the lingual gyrus in bipolar disorder patients [25]. Consequently, it was found that there was a significant positive correlation between the duration of illness and the resting-state functional connectivity of the right anterior temporal-parietal junction with the culmen and with the parahippocampus [25]. 

A study examining the functional connectivity of the cerebellum vermis to regions of the cerebrum involved in emotion regulation in bipolar disorder [26]. The study found connections with emotion and motor control regions such as the amygdala and posterior cingulate were significantly stronger in bipolar disorder, while regions associated with language production were significantly weaker [26]. Participants with bipolar disorder reporting lower mood ratings demonstrated vermis connectivity to the right anterior parahippocampal gyrus, which is involved with emotional salience, and the left middle frontal gyrus, which is involved with emotional regulation [26]. The authors concluded that these findings suggest a “potential compensatory role for the cerebellum associated with emotional regulations,” whereby different mood states are due to the vermis failing to compensate with different regions of the brain [26]. The authors hypothesize this could be due to differences in cerebellar metabolism in bipolar disorder [26]. Saleem et al. (2023) also found no effect of medication on these functional connectivities [26]. 

Discussion

The anatomical findings in bipolar disorder point to consistent alterations within the cerebellum, shedding light on its potential role in the pathophysiology of the disorder. Several studies have reported a pattern of reduced gray matter density in specific cerebellar regions, particularly in the right hemisphere. This includes lobules I-IV, V, crus I, and crus II, with the right cerebellar hemisphere appearing to be more affected. Interestingly, both bipolar I disorder and bipolar II disorder patients exhibit these gray matter changes, suggesting a shared cerebellar signature across different subtypes of bipolar disorder. Furthermore, bilateral involvement of cerebellar crus I and crus II has been consistently observed in bipolar disorder, underlining the significance of these regions in the disorder. These findings collectively suggest that cerebellar gray matter alterations may contribute to mood dysregulation and cognitive dysfunction in bipolar disorder.

Gray matter reductions in various cerebellar regions appear to be associated with the multifaceted symptomatology of bipolar disorder, encompassing motor and emotional symptoms as well as traits like impulsivity, disinhibition, hyperactivity, and cognitive empathy reduction. Notably, studies consistently reveal that bipolar disorder patients struggle with identifying social faux pas in narratives and comprehending others' behaviors and mental states. Additionally, bipolar II disorder individuals exhibit reduced accuracy in detecting negative mental states during the Reading the Mind in the Eyes test, potentially implicating the visual system in bipolar disorder’s neurobiological underpinnings. 

Regarding medication effects, the impact on cerebellar anatomy is mixed, emphasizing the need for in-depth investigations into medication-specific influences on cerebellar and cerebral anatomy, underlining the intricate interplay of medications in bipolar disorder.

Minichino et al. (2015) explored the effects of transcranial direct current stimulation (tDCS) on cognitive performance in euthymic bipolar disorder patients [27]. Transcranial direct current stimulation was administered with excitatory stimulation of the left dorsolateral prefrontal cortex and inhibitory stimulation of the right cerebellar hemisphere [27]. Results showed significant improvements in visuospatial memory, motor coordination, executive functioning, and neurological soft signs [27]. Stratifying patients based on baseline cognitive impairments revealed that those with more severe impairments demonstrated greater improvements [27]. Their study suggests that the modulation of prefrontal-cerebellar circuitry activity through tDCS may have potential therapeutic benefits for cognitive deficits in bipolar disorder patients [27]. 

The limitations across the mentioned articles collectively underscore several common shortcomings. Many studies had small sample sizes, limiting the generalizability and statistical power of their findings. Additionally, several of them employed a crossover study design, limiting the ability to establish causation, draw long-term conclusions, and determine if the anatomic findings seen in bipolar disorder are progressive in nature. Specific subtypes of bipolar disorder (bipolar I disorder and bipolar II disorder) were not always distinguished, potentially impacting the validity of the results. In some studies, participants with a history of alcohol, cannabis, or other psychoactive substance use, such as lorazepam, were not included, which may potentially confound the anatomical findings seen.

## Conclusions

In conclusion, the examined studies in this literature review provide valuable insights into the significant relationship between the cerebellum and bipolar disorder. The findings suggest that cerebellar gray matter alterations, particularly in regions such as lobules I-IX, crus I, and crus II, may contribute to mood dysregulation, cognitive dysfunction, and social cognitive impairments observed in bipolar disorder. However, it is also important to note the limitations of these studies, which underscores the need for larger, more diverse samples and prospective, longitudinal designs to advance our understanding of bipolar disorder and its relationship with the cerebellum. Nonetheless, the emerging role of the cerebellum in bipolar disorder offers potential opportunities for the development of more effective therapeutic interventions. By further investigating the role of the cerebellum in the pathophysiology of bipolar disorder, we can explore new possibilities for targeted treatment strategies that may improve the lives of individuals affected by this complex psychiatric condition.
